# Effect of tobacco and other habitual dietary staining agents on the optical properties of lithium disilicate molar crowns: A laboratory study

**DOI:** 10.18332/tid/208433

**Published:** 2025-09-09

**Authors:** Mohammed M. Al Moaleem, Arwa Daghrery, Heba Mitwalli, Eman Jabarti, Nassreen Albar, Maysaa Khojah, Waad Khayat

**Affiliations:** 1 Department of Prosthetic Dental Sciences, College of Dentistry, Jazan University, Jazan, Kingdom of Saudi Arabia; 2 Department of Restorative Dental Sciences, College of Dentistry, Jazan University, Jazan, Kingdom of Saudi Arabia; 3 Department of Restorative Dental Science, College of Dentistry, King Saud University, Riyadh, Kingdom of Saudi Arabia; 4 Dental Department, Dr Sulaiman Al Habib Medical Group, Riyadh, Kingdom of Saudi Arabia; 5 Department of Preventive Dentistry, Faculty of Dental Medicine, Umm Al-Qura University, Mecca, Kingdom of Saudi Arabia; 6 Department of Restorative Dentistry, College of Dental Medicine, Umm Al-Qura University, Mecca, Kingdom of Saudi Arabia

**Keywords:** color change, khat, lithium aluminum disilicate, Saudi coffee, snuff

## Abstract

**INTRODUCTION:**

The treatment of choice for posterior teeth is full-coverage crowns, but these materials should not be color-affected by external factors such as tobacco use. This laboratory study aimed to evaluate the mean color change (ΔE*) values of lithium aluminum disilicate (LAD) full anatomical ceramic crowns after staining in different adverse materials consumed in different countries worldwide.

**METHODS:**

Ninety full anatomical crowns in the form of molars were constructed from LAD (Cerec Tessera blocks) with the use of computer-aided design/computerassisted manufacturing system. They were divided equally into nine groups, with 10 crowns for each group. The crowns were immersed for 15 days in different adverse materials (khat, shamma, yerba mate, snuff, soft drinks used daily as a mixture of fruit juice, Coca-Cola, Saudi coffee, and Nescafe). Instructions from the International Commission on Illumination were followed for color parameter measurements. The CIELab color space L* lightness value coordinate, a* red– green coordinate, and b* yellow–blue coordinate, were calculated before and after staining for 2 weeks. ΔE* values were calculated by spectrophotometry. One-way ANOVA followed by *post hoc* tests were used to assess significance differences between groups. The significance level was set at p<0.05.

**RESULTS:**

The ΔE* changed after immersion in all solutions except for the control group. The highest average ΔE* values and standard deviation were observed with yerba mate 6.3 ± 2.0, followed by shammah 4.6 ± 0.9, whereas the lowest ΔE* was recorded for the mixture of fruit juice 3.2 ±1.3. One-way ANOVA test showed a significant difference between the group of yerba mate, with p<0.001. Pearson’s correlation coefficient test was used to assess the statistical relationship of ΔE* among groups. A highly positive significant correlation was found between shammah and Nescafe (r=0.798). A medium correlation was found between khat with yerba mate (r=0.520) and snuff and control groups (r=0.474), without significant differences.

**CONCLUSIONS:**

The overall recorded ΔE* values for LAD full anatomical ceramic crowns following 2 weeks of staining in different staining media were marginally higher than the clinically acceptable values except for the fruit-juice group.

## INTRODUCTION

Computer-aided design/computer-assisted manufacturing (CAD/CAM) technology has created a paradigm shift in the development of lithium disilicate glass-ceramics (LDGCs) with an array of compositions emerging in the market at an advanced rate^1^. This system expands the possibilities for fabricating crowns within fewer appointments^2^ and with high biocompatibility, exceptional aesthetics, and improved biomechanics beyond those offered by conventional techniques^3^.

Aesthetic dental practice has brought numerous innovative clinical procedures and a revolution in dental materials. LDGCs are the most commonly used glass ceramics because of their exceptional optical properties, superior strength, and ease of fabrication^4^. LDGCs include better marginal integrity, less porosity, and net-shaped formation by pressing^5^. LAD ceramic combines a high biaxial flexural strength of >700 MPa and aesthetically pleasing results^6^. The most recent LAD added to the market is commercially marketed as an advanced lithium disilicate glass-ceramic (CEREC Tessera, Dentsply Sirona, York, PA, USA), which contains LAD crystals known as virgilite within its glassy zirconia matrix^7^. It has been developed for chairside CAD-CAM systems to improve aesthetic and mechanical properties^8-10^.

The optical, biological, and mechanical properties of ceramics have vastly improved over time, but they remain susceptible to discoloration^11,12^. Various external factors, including exposure to different solid or liquid beverages, acidic solutions, tooth brushing, and high temperatures, can lead to surface degradation in dental prostheses^13-15^. The absorption or adsorption of extrinsic pigments from the oral cavity depends on the composition and surface morphology of the ceramic materials^16^. Numerous discolorants are part of daily life for people around the world; these include coffee, tea, and sports drinks. Additionally, khat, a green leaf that naturally stimulates the central nervous system, is chewed by around 5 million people globally. This practice is particularly prevalent in Yemen, certain regions of Africa, and southern Saudi Arabia^17^.

In recent years, global tobacco demand has surged. The World Health Organization estimates that the number of tobacco users now exceeds 1.3 billion people^18^. Another beverage of interest is yerba mate, made from the leaves of the Ilex tree (*Mate folium*). After the leaves are dried and roasted, they are used to prepare the Paraguayan tea known as ‘yerba mate’. About 450 species of Ilex grow in the tropical regions of South America and Asia, and yerba mate is exclusively consumed in South America, particularly in northern Argentina, southern Brazil, Uruguay, and Paraguay^19^.

Many studies assessed the effects of different habits individually or combined, such as khat, smokeless tobacco, yerba mate, smokeless snuff, and other soft drinks used daily (e.g. Coca-Cola, Nescafe, and Saudi coffee) and revealed variable results^12,20^. Previous studies typically assessed ceramic discs by immersion in staining solutions, exposing the specimens to staining from both sides^13,18^. This method does not accurately reflect real clinical situations. Furthermore, the thickness of the ceramic discs used in these studies is greater than that of actual ceramic restorations used in practice. Using crown-shaped specimens for color stainability studies more accurately represents clinical scenarios, providing more consistent and standardized measurements^13^.

Methods for evaluating light reflectance, light transmittance, and color have been reported to understand the phenomena that occur when light strikes an object^21^. The *Commission Internationale de l’Eclairage* (CIE) introduces the main color systems, mean color-change concepts, and illumination patterns used in science^21^. The CIELab system defines mean color change (ΔE*) as the standard parameter for color-matching perception^21^. The CIELab color space (L*: lightness value coordinate; a*: red–green coordinate; b*: yellow–blue coordinate) neglects other components and factors on color perception, such as translucency, opalescence, fluorescence, and surface texture^22^.

Daily habits are influenced by individual preferences and vary significantly across societies. In this study, we comprehensively compared various social habits practiced globally and rigorously evaluated their impact on the optical properties of LAD ceramics. Thus, the present study aimed to evaluate the mean color change (ΔE*) of LAD ceramic full anatomical crowns following two weeks of staining in different adverse materials consumed in different countries worldwide (khat, smokeless tobacco/shammah, yerba mate, smokeless snuff, and other soft drinks used daily, such as mixture of fruit juice, CocaCola, Nescafe, and Saudi coffee). The primary null hypothesis was that no significant difference existed in the ΔE* of LAD ceramic full anatomical crowns after staining with different daily consumed products; the secondary null hypothesis was that no significant differences in ΔE* existed between all tested products.

## METHODS

### Study design and ethical approval

Within this experimental study, the effect of different adverse social habits on the ΔE* of LAD was assessed. A total of 90 CAD/CAM LAD anatomical crowns for molars teeth were fabricated. Supplementary file Table 1 presents the materials and devices used. This work was approved by the Ethics Research Committee at Jazan University (Rec-44/07/496).

### Sample size calculation

The crown number size was calculated using G*Power software (version 3.1.9.4, University of Dusseldorf). The effect size (d) was 0.5, α was 0.05, and 1-β (power) was 0.80. The sample size obtained was 90 crowns divided equally among nine social habit groups for detecting a large effect size (0.40) with a 1-β (power) of 0.80, and it was considered in accordance with the mean color change of the color from earlier published studies^11^.

### Teeth preparations

Ninety sound maxillary molar teeth were collected from different clinics with sound clinical crowns and extracted owing to periodontal causes. The extracted teeth had equal average crown dimensions. For the allceramic crown’s preparation, the occlusal preparation was 1.5 ± 0.25 mm, the axial wall preparation was 1.0 ± 0.25 mm, and the shoulder finish line was 1.0 ± 0.25 mm in width as recommended by the manufacturer and textbook.

### Samples manufacturing and grouping

All prepared teeth were mounted on acrylic resin dye then scanned using the CAM-machine CEREC Primemill (THE DENTAL SOLUSIONS COMPANY, Dentsply Sirona, Germany). The anatomical crowns were constructed from CEREC Tessera Advanced LAD (THE DENTAL SOLUSIONS COMPANY, Dentsply Sirona, Germany). The color used was HT A2 C14. The cutting dimensions of the specimens were determined, considering that a 0.2–0.3% shrinkage was encountered during LAD crystallization. The fully anatomical crowns were 2.0 ± 0.2 mm thick on the occlusal surfaces (area of color-parameter measurements), measured using a digital Vernier caliper (ABSOLUTE DIGIMATIC 0–150 mm, Mitutoyo Digital, Japan).

The samples were consecutively polished with 600, 800, and 1000 grit silicon carbide papers with water for LAD crowns as recommended by the manufacturers. Each polishing step was performed for 60 s at 300 rpm by one single operator^23^. Afterwards, the LAD full anatomical crowns were conventionally crystallized in a furnace (CEREC Speed Fire Furnace, THE DENTAL SOLUSIONS COMPANY, Dentsply Sirona, Germany). The samples were then placed in sterilization sealing packs, separated from one another by using separators, and randomly divided into nine groups (10=/group) in accordance with the staining material type.

### Color parameters measurements

The HT A2 C14 shade served as the standard shade for all samples. After undergoing polishing and crystallization, the samples were cleaned in an ultrasonic bath of 99% isopropyl alcohol for 5 min before proceeding with the measurements. The spectrophotometer Vita Easyshade Compact (Vita Zahnfabrik H. Rauter GmbH & Co. KG, Bad Säckingen, Germany) version V with a 4 mm diameter tip and a calibration plate was used to record the color parameters by using the CIE L*a*b* at the center of each LAD anatomical crown. The wavelength used was 555 nm, because the human eye is sensitive to wavelengths ranging between 380 and 780 nm (the highest sensitivity is at 555 nm)^16^. This wavelength was selected on the basis of the definition of CIE^21^. The optical handpiece was held at a 90° angle to the disks with no incandescent lights. A probe tip was inserted into the calibration port aperture of the device after each measurement to enable the calibration of the device for each sample, as recommended by the manufacturer^12,16^.

**Figure 1:**
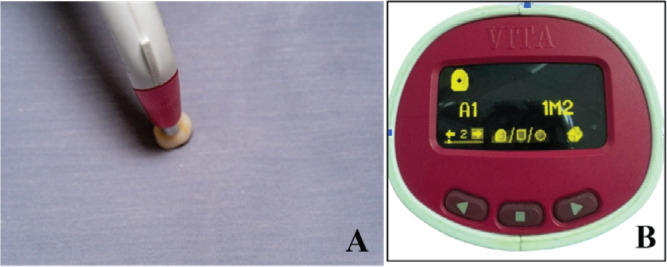
During color parameter measurements over gray background (A), reading after measurements with VitaPan classical shade guide (B)

After the anatomical crowns were carried from the laboratory, they were cleaned, and the spectrometric device was calibrated. All anatomical crowns for each group were filled with gray modeling clay (MODELING CLAY, Class, Belgium), and then the first readings of color parameters (baseline values L1, a1, and b1) were recorded over a gray background (Figure 1).

The recorded values were used to calculate ΔE*, which was defined as the difference between two colors in an L*a*b* color space. Its value was evaluated by calculating the difference in color measurements of the crowns before and after 2 weeks of staining with 50%:50% of the perceptibility and acceptability threshold and with a mean color change (ΔE*) values between 1.7 and 4.2^20^ as follows ^12,16^:

ΔE* = [(L1* - L2*)^2^ + (a1* - a2*)^2^ + (b1* - b2*)^2^]^1/2^

where L1, a1, and b1 are the color data measured before staining (baseline) with social habits materials, and L2, a2, and b2 are the color data following immersion for 2 weeks in different social-habit beverage materials. The chromatic values for the assessed anatomical LAD crowns were measured on the center of each crown three times with the device, and then the averages were recorded.

### VitaPan classical shade guide assessments

The LAD anatomical crowns were assessed using the VitaPan classical shade guide (Vita Zahnfabrik H. Rauter GmbH & Co. KG, Bad Säckingen, Germany). The procedure was performed by a blindtrained operator before the crowns were subjected to immersion in different staining media. These assessments were considered as ‘before’. They were carried out against a gray background to mimic the non-existence of light in the oral cavity, as shown in Figure 1. This action was conducted in accordance with the VitaPan shade guide ISO/TR 28642: 2016. Then, after 2 weeks of immersion and staining with different staining materials, the color of different groups was measured, and the differences were recorded as unchanged, changed into darker, or changed into lighter colors.

### Immersion of anatomical crowns in the different staining materials

Khat was prepared and offered by the Substance Abuse and Toxicology Research Center at Jazan University. The homogenate of khat leaves was prepared from fresh minced fresh khat leaves in 100% distilled water (V/W) and finely minced. The khat was then kept in a -80°C ultralow-temperature freezer until use. It was mixed with NaOH until its pH was similar to the pH of saliva and the oral cavity. Only 10 anatomical crowns were stained in khat for 15 days as mentioned in previous *in vitro* studies^15,24^. The same procedures were executed daily to obtain fresh solutions.

All anatomical LAD crowns were stained in black smokeless tobacco (shammah) for 2 weeks, as reported earlier^18^. The black shammah sample was mixed with water to a thick consistency. The crowns were immersed for 10 min and with a constant weight bearing down to ensure the crowns remained immersed for 10 min (the actual using time for shammah) to simulate the force generated during dipping of shammah. The shammah preparations or blouse were replaced two times daily in accordance with the actual usage^25^. The smokeless snuff came with a covering bed, and it was ready to be dissolved in saliva after placing it in the buccal sulcus. For this group, the crowns were immersed between the snuff and covered on the top. The crowns were immersed as performed for shammah in relation to immersion time, and the load over crowns and snuff was constant. The snuffs were replaced two times daily in accordance with the actual usage^26^.

Yerba mate tea staining solutions were prepared by adding 15 g – 250 mL of boiled distilled water, and then boiled water was added after 10 min to refresh the homogeneity of the drink. The powder materials were changed every 12 h daily^19,27^. The coffee (Nescafe Classic, Nestle Middle East Manufacturing LLC, Dubai)-staining solution was prepared by adding 15 g – 250 mL of boiled distilled water. The test solution was changed daily and stirred once every 12 h to maintain the homogeneity of the solution^12,13^.

Following the baseline color assessments, 10 anatomical crowns were stained in Saudi coffee (Baja Food Industrial Co., Jeddah, Saudi Arabia) during the aging period. The instant Saudi coffee with cardamom came in a nitrogen-flushed packet for single use. The staining solution prepared from each packet (30 g) was mixed with 0.5 L of boiled water at 100°C and kept boiling for 15 s. Coffee solutions were changed every 12 h11,15,28.

For the fruit juice mixture, 10 of the anatomical LAD crowns were immersed in solutions completely in a vertical position. Coca-Cola was ready to use and changed every 12 h^29^. The control group was stained and immersed in normal saline directly. One trained operator (Al M M) performed all the color measurements.

**Figure 2:**
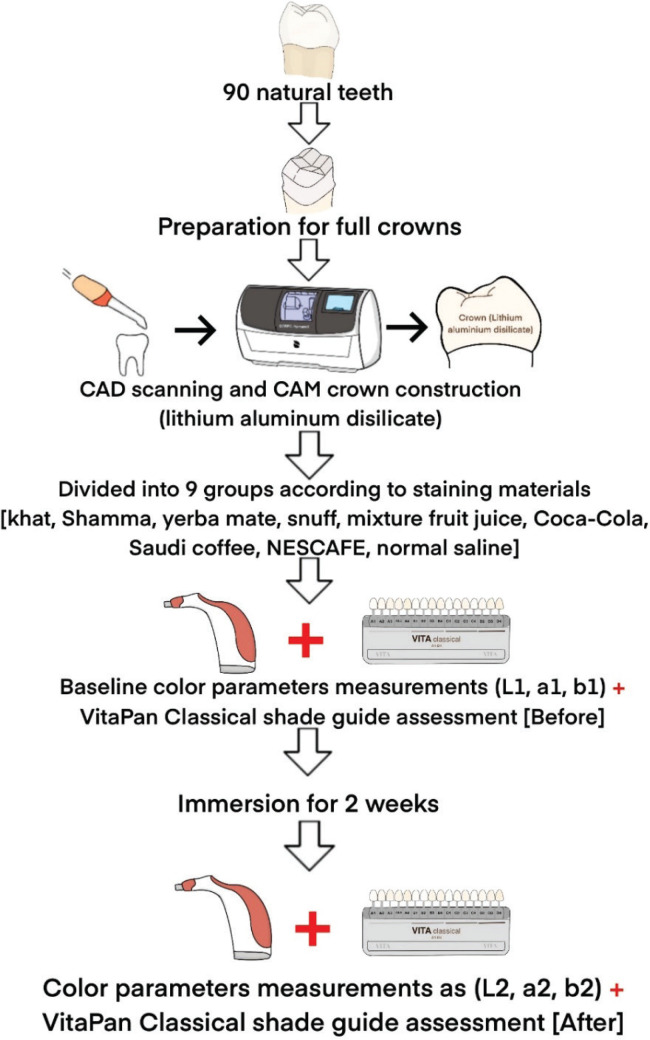
The flowchart of steps included in this study

All staining media, materials, and solutions were shaken well before actual use. A 12 h refreshment of solutions was conducted. Each group was stained in a separate container and kept inside an incubator during the immersion period. After immersion for 15 days, the anatomical crowns were removed from the test solution. During the staining period and aging process (storing the crowns with their staining materials inside an incubator at 37°C), the nine groups of crowns were dipped in distilled water 10 times, wiped with tissue paper, and left to completely air dry. Figure 2 represents the flowchart of steps included in this study.

### Statistical analysis

The mean and standard deviation of the color change ΔE* of the full contour crowns were recorded before and after immersion in nine different social-habit beverages and then aged for 2 weeks. SPSS (version 26.0, SPSS Inc., Chicago IL, USA) was used to input and analyze the data. One-way ANOVA and paired Student’s t-tests were applied to detect any significant difference between groups.

Most of the data were normally distributed by the Shapiro-Wilk test (p>0.05). One-way ANOVA was applied to detect any significant difference between the groups. A *post hoc* Tukey HSD test was used for comparing between every two groups. Pearson’s correlation coefficient test was used to assess the statistical relationship between the average ΔE*. A p<0.05 was considered as a cutoff point for statistical significance.

## RESULTS

Table 1 shows the mean values of ΔL, Δa, and Δb before and after immersion in nine different discoloring and staining solutions. All mean values of L, a, and b changed (either decreased or increased) after immersion in all discoloring solutions, except for smokeless tobacco (snuff), where L and b remained unchanged.

**Table 1:** Color parameter values of lithium disilicate molar crowns before and after immersion in staining solutions

*Staining media*	*Interval*	*ΔL*	*Δa*	*Δb*
		*Mean ± SD*	*Mean ± SD*	*Mean ± SD*
Khat	Before	77.8 ± 2.1	-2.2 ± 0.7	19.8 ± 3.9
	After	75.4 ± 2.5	-1.5 ± 0.9	21.9 ± 2.4
Shammah	Before	75.5 ±1.1	-3.4 ± 0.9	17.3 ± 3.5
	After	73.6 ± 2.9	-1.8 ± 0.8	25.6 ± 5.4
Yerba mate	Before	78.8 ± 2.0	-2.0 ± 0.6	21.1 ± 3.3
	After	77.7 ± 1.4	-2.6 ± 0.5	27.2 ± 4.0
Snuff	Before	76.4 ± 1.3	-2.4 ± 0.5	20.8 ± 4.2
	After	76.6 ± 3.9	-2.2 ± 0.6	20.7 ± 4.1
Mixture of fruit juice	Before	77.2 ± 2.0	-2.8 ± 0.5	18.3 ± 3.9
	After	74.6 ± 2.2	-1.4 ± 0.7	16.5 ± 2.2
Cola	Before	78.7 ± 2.3	-2.7 ± 0.7	20.7 ± 5.0
	After	79.0 ± 4.0	-1.7 ± 0.4	21.0 ± 3.1
Nescafe	Before	76.7 ± 1.8	-3.4 ± 0.9	19.0 ± 4.2
	After	76.4 ± 2.6	-1.7 ± 1.1	18.6 ± 5.4
Saudi coffee	Before	76.5 ± 1.6	-1.9 ± 0.7	19.3 ± 5.6
	After	77.0 ± 0.9	-2.2 ± 0.6	17.8 ± 5.4
Control	Before	76.1 ± 1.7	-2.2 ± 0.9	20.0 ± 4.7
	After	75.1 ± 3.4	-2.2 ± 0.8	17.1 ± 2.6

CIELab color space: L*: lightness value coordinate; a*: red–green coordinate; b*: yellow–blue coordinate.

**Figure 3:**
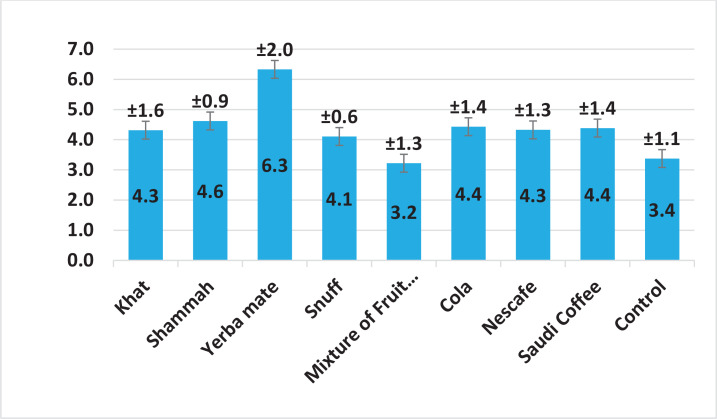
Mean *Δ*E* (± SD) values for different staining solutions

Figure 3 show that the highest average ΔE* values and standard deviation were obtained in yerba mate solution 6.3 ± 2.0, followed by shammah 4.6 ± 0.9, after immersion and staining using the different materials. The lowest ΔE* values were recorded for the mixture of fruit juice 3.2 ±1.3.

ANOVA also showed a significant difference in the average color change of ΔE* between groups of social habits (p=0.0002, Table 2). The *post hoc* Tukey HSD test was used for comparing between different social habits indicated statistically significant differences in the average color change of ΔE* between yerba mate and khat, snuff, mixture of fruit juice, Nescafe and the control group (p=0.0300, 0.0104, <0.0001, 0.0315, and 0.0001, respectively), as shown in Table 3.

**Table 2:** Comparison^a^ of the color change of *Δ*E* between exposure groups among lithium disilicate molar crowns

*Staining media*	ΔE* *Mean ± SD*
Khat	4.3 ± 1.6
Shammah	4.6 ± 0.9
Yerba mate	6.3 ± 2.3
Snuff	4.1 ± 0.6
Mixture of fruit juice	3.2 ± 1.3
Cola	4.4 ± 1.4
Nescafe	4.3 ± 1.3
Saudi coffee	4.4 ± 1.4
Control	3.4 ± 1.1

a ANOVA test: df=8, mean square=7.863, F=4.400, p=0.002 (significant).

**Table 3:** Pairwise comparisons^a^ between different exposures of lithium disilicate molar crowns

*Discoloring solutions*		*Difference*	*SE*	*p*
**Khat**	Shammah	0.3	0.59785	0.999
	Yerba mate	2.0	0.59785	0.030*
	Snuff	0.2	0.59785	1.000
	Mixture of fruit juice	1.1	0.59785	0.661
	Cola	0.1	0.59785	1.000
	Nescafe	0.0	0.59785	1.000
	Saudi coffee	0.1	0.59785	1.000
	Control	0.9	0.59785	0.817
**Shammah**	Yerba mate	1.7	0.59785	0.114
	Snuff	0.5	0.59785	0.994
	Mixture of fruit juice	1.4	0.59785	0.330
	Cola	0.2	0.59785	1.000
	Nescafe	0.3	0.59785	0.999
	Saudi coffee	0.2	0.59785	1.000
	Control	1.2	0.59785	0.492
**Yerba mate**	Snuff	2.2	0.59785	0.010*
	Mixture of fruit juice	3.1	0.59785	<0.001*
	Cola	1.9	0.59785	0.051
	Nescafe	2.0	0.59785	0.031*
	Saudi coffee	1.9	0.59785	0.042*
	Control	2.9	0.59785	<0.001*
**Snuff**	Mixture of fruit juice	0.9	0.59785	0.861
	Cola	0.3	0.59785	0.999
	Nescafe	0.2	0.59785	1.000
	Saudi coffee	0.3	0.59785	0.999
	Control	0.7	0.59785	0.949
**Mixture of fruit juice**	Cola	1.2	0.59785	0.532
	Nescafe	1.1	0.59785	0.650
	Saudi coffee	1.2	0.59785	0.5828
	Control	0.2	0.59785	1.000
**Cola**	Nescafe	0.1	0.59785	1.000
	Saudi coffee	0.0	0.59785	1.000
	Control	1.1	0.59785	0.705
**Nescafe**	Saudi coffee	0.1	0.59785	1.000
	Control	1.0	0.59785	0.808
**Saudi coffee**	Control	1.0	0.59785	0.751

a *Post hoc* Tukey HSD test. SE: standard error.

* Significant.

Pearson’s correlation coefficient test was used to assess the statistical relationship among the ΔE* values recorded. A high positive significant correlation existed between shammah and Nescafe (r=0.798), whereas a moderate correlation existed between khat and yerba mate (r=0.520) and between snuff and the control group (r=0.474), but without significant differences. Meanwhile, a negative moderate correlation was observed between yerba mate and shamma and Nescafe (r= -0.553 and -0.500, respectively), but this correlation was not significant as noted in Table 4.

**Table 4:** Correlation between the difference in color changes of lithium disilicate molar crowns recorded by *Δ*E* in different discoloring solutions

*Discoloring solutions*		*Khat*	*Shammah*	*Yerba mate*	*Snuff*	*Mixture of fruitjuice*	*Cola*	*Nescafe*	*Saudi coffee*	*Control*
**Khat**	Pearson correlation	1								
	p									
**Shammah**	Pearson correlation	-0.242	1							
	p	0.501								
**Yerba mate**	Pearson correlation	0.520	-0.553	1						
	p	0.123	0.097							
**Snuff**	Pearson correlation	0.174	0.013	0.015	1					
	p	0.630	0.971	0.967						
**Mixture of fruit juice**	Pearson correlation	0.300	-0.387	0.139	-0.073	1				
	p	0.399	0.269	0.702	0.841					
**Cola**	Pearson correlation	-0.049	0.358	-0.393	0.259	0.400	1			
	p	0.893	0.310	0.261	0.471	0.252				
**Nescafe**	Pearson correlation	-0.051	0.798**	-0.500	-0.135	-0.140	0.315	1		
	p	0.889	0.006	0.142	0.709	0.699	0.376			
**Saudi coffee**	Pearson correlation	-0.399	0.143	0.004	0.152	-0.123	-0.049	0.201	1	
	p	0.253	0.694	0.990	0.675	0.734	0.893	0.578		
**Control**	Pearson Correlation	0.162	0.342	-0.623	0.474	-0.064	0.480	0.427	-0.036	1
	p	0.656	0.333	0.054	0.166	0.860	0.161	0.219	0.921	

**Figure 4:**
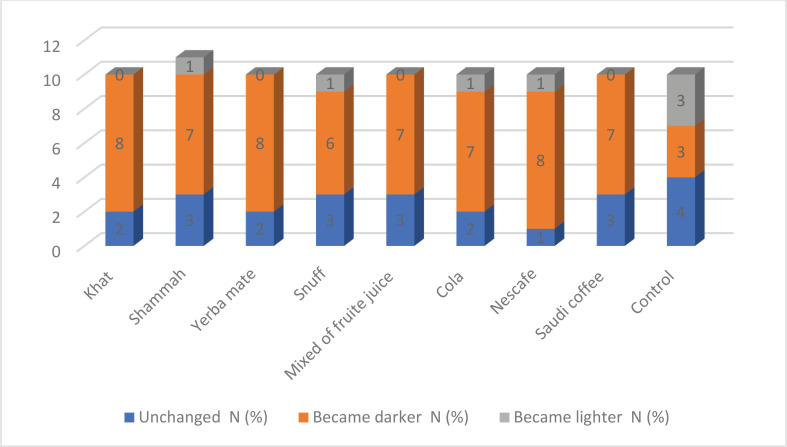
Number of sample changes in VitaPan classic shade guide after immersion for 2 weeks

Figure 4 shows that in relation to the VitaPan classical shade guide, most anatomical crowns darkened after immersion for 2 weeks in different staining media. The highest percentages were recorded in crowns stained with khat, yerba mate, and Nescafe (8 samples out of 10), followed by shammah, mixture of fruit juice, Coca-Cola, and Saudi coffee (7 samples out of 10).

## DISCUSSION

Full coverage crowns on the molar teeth remain the choice in daily dental office practice. These crowns should maintain their color over a period of time in the presence of different social habits. This study aimed to evaluate the values of ΔE* and VitaPan classical shade guide changes om LAD ceramic anatomical crowns after 2 weeks of staining in different adverse materials consumed in different countries worldwide (khat, shammah, yerba mate, snuff, and other soft drinks used daily, such as mixture of fruit juice, CocaCola, Nescafe, and Saudi coffee). The two-week *in vitro* staining period is equivalent to more than 14 months *in vivo* ^28,29^. In this study, posterior crowns were chosen because most daily habits related to social consumption occur in the posterior area of the mouth. Additionally, samples that reflect full anatomy can better represent actual clinical scenarios for crowns.

### Mean color change

The overall ΔE* values were considered slightly higher than the acceptable values and were between 3.4 ± 1.1 and 6.3 ± 2.3. According to Paravina et al.^30^, these values can be considered as moderately unacceptable (ΔE* ≤3.6) for the group of mixture of fruit juices; obviously unacceptable (ΔE* from >3.6 to ≤5.4) for the groups of khat, shammah, snuff, Coca-Cola, Nescafe, and Saudi coffee; or severely unacceptable for the anatomical crown group stained by yerba mate (ΔE* >5.4)^30^. Other studies have stated that the clinically acceptable color change values range from 1.7 to 3.7, but higher values can be considered as marginally unacceptable^20,31^. These values are much higher than those found in previous studies recording the ΔE* among anatomical crowns with LAD materials for incisor teeth^13^ and molar teeth^16^.

The primary null hypothesis stated that no significant difference existed in the ΔE* and LAD ceramic anatomical crowns after staining with different social habits; this is partially accepted as no significant differences were recorded between the different staining media, and because a single group that was the mixture of fruit juices recorded a significant difference from the other groups.

In this study, the recorded ΔE* values for LAD crowns after staining in different staining materials with LAD materials were higher than those recorded for anterior anatomical crowns by^13^. Also, it was higher than those recorded ΔE* by Kordi et al.^13^, those recorded ΔE* for molar teeth for the same brand material. The main reasons for the disagreements can be related to the location of ΔE* values measurements, which were performed on the occlusal surface in the present study, whereas others were carried out on the labial surface of the anterior teeth and the axial surface^16^, which is considered a flat surface. This difference played a role in the thickness of ceramic materials, presence of fissures and grooves, and cusp height. Furthermore, a minor change in the composition of the materials and immersion materials may play a role in the ΔE* values. The color of LAD ceramic full anatomical crowns is considered interesting for monolithic restorations produced for a digital workflow after a short period of follow-up^6^.

The effects of different social habits and materials used in the fabrication of dental prosthesis remain unknown. The discussion was divided in relation to staining social-habit materials: those that stayed; were chewed; and were kept in the mouth for minutes (khat, shammah, snuff, and yerba mate) or others, such as liquids or drinks that are swallowed directly through the mouth (Cola, Nescafe, Saudi coffee, and fruit juice).

Green khat leaves are chewed and kept in buccal vestibules as unilateral or bilateral in the form of bolus for at least 3–5 h^32^. Many different compounds are found in khat, including alkaloids, terpenoids, flavonoids, sterols, glycosides, tannins, amino acids, vitamins, and minerals. Phenylalkylamines and catha edulis are major alkaloids structurally related to amphetamine^32^. Khat shows acceptable changes in ΔE* for CAD/CAM porcelain in the form of polished or glazed surfaces^14^.

Oral use of smokeless tobacco is common in Africa, North America, Southeast Asia, Europe, and the Middle East. It comprises placing a piece of tobacco or tobacco product in the mandibular groove and either chewing or sucking it for a certain period of time^17,32^. The major components of smokelesstobacco products are becoming increasingly popular in Sweden, the UK, and the US, and these countries are determined to provide a greater understanding of the general chemical composition of these products^33^. A previous study has found that the ΔE* is between 2.7 and 4.8 for different CAD/CAM ceramic materials after immersion in smokeless tobacco^18^. The higher ΔE* values documented in the present study for groups stained with khat or shammah demonstrated the adverse effect of chewing such materials on the anatomical crowns compared with ceramic in the form of disk.

The Paraguayan tea known as yerba mate is composed of many species growing in certain countries, especially South America, Syria, and Lebanon^19^. After 14 days or 14 months of clinical staining, clinically acceptable data for cola and higher values for yerba mate tea were recorded. Clinically acceptable values were found after immersion in bisacryl resins^27^. The values that did not agree with the present study’s findings, may be due to the different compositions and forms of the stained restorative materials.

Soft drinks in cold or hot form, such as CocaCola, fruit juice, Nescafe, and Saudi coffee, that are swallowed or drank immediately had different levels of stainability on the color of CAD/CAM materials used for the construction of crowns. Haralur et al.^11^ recorded mean color changes of 1.79 and 2.24 for coffee and tea after immersion in LDGC. The highly aesthetic LDGC IPS e.max CAD recorded a ΔE* mean color change of 1.84 after thermal aging and staining for 15 days^34^. The values of ΔE* recorded for different ceramic materials after immersion with Saudi coffee were between 1.96 and 3.07^13,28^. Alghazali et al.^15^ recorded ΔE* values of 1.58 and 1.9 for e.max CAD and e.max Press, respectively, after immersion in Arabic qahwa. All of the abovementioned values were lower than the ΔE* values in the current study in all groups, which can be explained by the sample shape that were anatomical crowns compared with the disks in other studies.

The spectrophotometer device, with its values for L, a, and b, can be used to assess all or most dental restorative and prosthetic materials. Thus, this device is commonly recommended for use as a reference device in numerous studies on color-change measurements^35^. Many researchers have recorded the ΔE* and obtained accurate values for testing the stainability of different beverages^11,12,16,28,30^.

### Vita classical shade guide changes

The VitaPan classical shade guide serves to accurately determine tooth shade. It comprises four groups as follows: A1–A4 (reddish-brownish), B1–B4 (reddishyellowish), C1–C4 (grayish shades), and D2–D4 (reddish-gray). The secondary null hypothesis in relation to the VitaPan classical shade guide, most samples per group changed to a darker color, so the hypothesis was partially rejected.

According to the VitaPan classical shade guide, most anatomical crowns darken after staining in different social habits materials and beverages (Figure 4). This finding disagreed with a previous one because of the immersion of different types of ceramic materials (Vita Marc II, Vita Suprinity, and Vita Enamic) with shamma and thermocycling^36^. Almost identical results were recorded after staining different types of CAD/CAM ceramic disks (16 mm wide and 2.0 ± 0.1 mm thick) with Saudi coffee for different periods. Al Moaleem et al.^18^ and Adawi et al.^28^ documented that most tested ceramic disks darkened after smokeless tobacco immersion with different materials.

### Strengths and limitations

This study utilized a full-anatomical crown that mimics the type cemented in a patient’s mouth. Various discoloration materials used globally were tested in this laboratory experiment. Future clinical and laboratory research should explore different types of ceramic materials and their optical properties, as well as extending the immersion time in staining materials.

A single material, an advanced LAD CAD/CAM ceramic, was used to fabricate a single full anatomical crown. It can be considered as a limitation of this study. This crown was also used as a molar anatomical tooth, which was greater in size than other anatomical anterior and posterior teeth. Also, sample size, non-adjusted comparison, and residual confounding can be considered as limitations of the study. Different advanced prosthetic materials can be suggested for use in these types of social habits with a longer period of staining. The absence of an oral background and environment in the mouth, such as different temperatures, oral hygiene, and saliva, can be considered also as a limitation.

## CONCLUSIONS

Within the limitation of this *in vitro* study, the following conclusions can be drawn. The overall ΔE* values were considered slightly higher than the clinically acceptable values (4.2) for the tested materials and staining media. The average color change was considered moderately unacceptable for the group of mixtures of fruit juices; obviously unacceptable for the groups of khat, shammah, snuff, Coca-Cola, Nescafe, and Saudi coffee; or severely unacceptable for the anatomical crown group of yerba mate. Moreover, the 2-week immersion resulted in a darker shade according to the VitaPan classical shade guide.

## Supplementary Material



## Data Availability

The data supporting this research are available from the authors on reasonable request.
