# Supplementary material for: Effect of tobacco and other habitual dietary staining agents on the optical properties of lithium disilicate molar crowns: A laboratory study
Source: Tob Induc Dis. 2025 Sep 9;23:10.18332/tid/208433. doi: 10.18332/tid/208433 (PMC12418941; doi:10.18332/tid/208433)
Supplement: Supplementary file 1 [file TID-23-127-s1.pdf]

Supplementary Table 1. Materials and devices used in the study.

Material/Device Type	Type (Brand name)	Composition	Manufacturers/ Lot #	Color/Application per day
Highly esthetic - Lithium Aluminium Disilicate	CEREC Tessera Dentsply Sirona	90% $Li_2Si_2O_5$, 5% Li_3PO_4, 5% $Li_{0.5}Al_{0.5}Si_{2.5}O_6$	Tessera Dentsply Sirona (Germany)/ CE 0124	HT A2 C14
Khat or Qat	Catha edulis plant	Alkaloids, terpenoids, flavonoids, sterols, glycosides, tannins, amino acids, vitamins, minerals.	Substance Abuse and Toxicology Research Center at Jazan University as green leaves	Green/2
Smokeless tobacco (Shamma)	Black Shamma	largely manufactured by powdering the tobacco along with ash, flavors, oils, calcium oxide, and black pepper	We get it from the market in a plastic package.	Black/ 2
YERBA MATE	ENVASADA ENORIGEN TARAGUI	caffeic acid, caffeine, caffeoyl derivatives, caffeoylshikimic acid, chlorogenic acid, feruloylquinic acid, kaempferol, quercetin, quinic acid, rutin, and theobromine	INDUSTERIA ARGENTEINA/ 7 790387 100310	Green/ 2
Smokeless tobacco (Snuff)	Snus	Water. With Swedish portion snus water and pouch material comprise more than half of the product mass; with chewing tobaccos water and sugars comprise around 60% of the products. With these STPs, tobacco was a minor component (30–35%) of the product mass	Snus, Sweden/ B.NO. 230600118<br>MFG. 10/2023	White/2
Mixture of fruit juice	Almarai	blending different fruits – pineapple, grapes, orange, strawberry, apple together. It is loaded with vitamins, minerals, antioxidants and thus benefits to boost the immune system of the body.	Almarai, Saudi Arabia	Red/2
Coca-Cola	Coca-Cola	Sugar, caramel, caffeine, orthophosphoric acid, water	Coca-Cola, SA	Black/2
NESCAFE<br>3 in 1 (STRONG)	NESCAFE	Sugar, glucose syrup, instant coffee (11%), palm kernel oil, soluble fibre, skimmed MILK powder (0.7%), MILK protein, salt, stabilisers, lactose (MILK), acidity regulator, emulsifiers, natural flavourings, MILK fat, colour	Nescafe, Saudi Arabia/ 31877900E1 A 12;54. Product-Date 07/2023, Exp 09/2024	Black/2
Saudi Coffee Mix (Cardamom)	Baja	Instant Saudi Coffee, Cardamom, Cloves, Nondaily Coffee Creamer, Saffron, used as Hot Coffee	(Baja Food Industrial Co., Jeddah, Saudi Arabia)/ 6281105795433 Product-Date16/ Jan/2023, Exp 16/10/2025	Yellowish/2
Spectrophotometer	VITA Easyshade® V	Device used to measure wavelength transmitted from one object at a time, without being affected by subjective interferences of color	VITA Zahnfabrik H. Rauter GmbH & Co. KG, Bad Sackingen, Germany/ 10180	Device for color parameters L, a, b measurements.

Vitapan classical shade guide	VITA classical A1-D4® shade guide	Used to assess the shade guide of teeth	VITA Zahnfabrik H. Rauter GmbH & Co. KG, Bad Säckingen, Germany)/ Prod. no. G027C	Shade matching kit

© 2025 Al Moaleem M.M. et al.
